# Multiple sites of soft-tissue metastases secondary to lung cancer: A case report

**DOI:** 10.1097/MD.0000000000018162

**Published:** 2019-12-10

**Authors:** Xingxing Zhu, Jialu Chen, Fanfan Yang, Congsheng Tang

**Affiliations:** a Department of Respiratory Therapy; b Department of Gynaecology, Women's & Children's Hospital of Haining city; c Department of Pathology, People's Hospital of Haining city, Haining, Zhejiang province, China.

**Keywords:** biopsy, lung cancer, PET-CT, soft tissues metastasis

## Abstract

**Rationale:**

The prognosis of lung cancer is dismal, which has resulted in lung carcinoma being one of the leading causes of cancer-related deaths worldwide. Non-small cell lung cancer accounts for approximately 80% of all types of lung carcinoma. The skeletal system and central nervous system are the most common distal metastatic sites in patients with lung cancer, while cutaneous and soft tissues metastasis is rare.

**Patient concerns:**

We report a case of concomitant metastasis in the nasal tip and suspected buttocks metastasis secondary to lung cancer, who complained of repeated cough and white sputum for 6 months.

**Diagnose:**

Primary lung cancer was diagnosed by bronchoscopy and biopsy, lesion on nasal tip was confirmed by biopsy. Furthermore, PET-CT scan identified the untouchable buttocks lesion that could have been easily missed.

**Interventions:**

This patient refused systemic treatments, but he chose traditional Chinese medicine at home.

**Outcomes:**

He died 6 months after the diagnosis.

**Lessons:**

The possibility of metastasis of primary cancers should be considered when encountering soft-tissue neoplasm lesions, and a biopsy of the suspicious cutaneous lesions could likely aid in the histological identification of the primary cancer. PET-CT scan could be an effective supplementary tool for the diagnosis and evaluation of cancers.

## 1 Introduction

Lung cancer remain a focus of the public, patients, physicians, and health authorities owing to its increasing incidence worldwide, along with the concomitant increase in morbidity and mortality.^[1]^ The prognosis of lung cancer is comparatively worse than most other others, primarily because of delayed diagnosis and early metastasis^[2]^; this has also resulted in lung carcinoma being the leading cause of cancer-related deaths.^[3,4]^ Non-small cell lung cancer (NSCLC) accounted for approximately 80% of all lung cancer types.^[5]^ Based on a large-scale investigation on patients with NSCLC, the following preferential metastasis sites were observed: bone, 34.3%; lung, 32.1%; brain, 28.4%; adrenal glands, 16.7%; liver, 13.4%; and extrathoracic lymph node, 9.5%.^[6]^ Even though the total incidence of skin metastasis was only about 1%, the rate of skin metastasis was second highest in lung cancer, secondary to internal malignancies.^[7,8]^ However, reports regarding multiple sites of soft tissues metastasis are very rare. Here, we report a case wherein concomitant nasal tip and buttock metastases were observed secondary to lung cancer.

## 2 Case presentation

**Figure 1:**
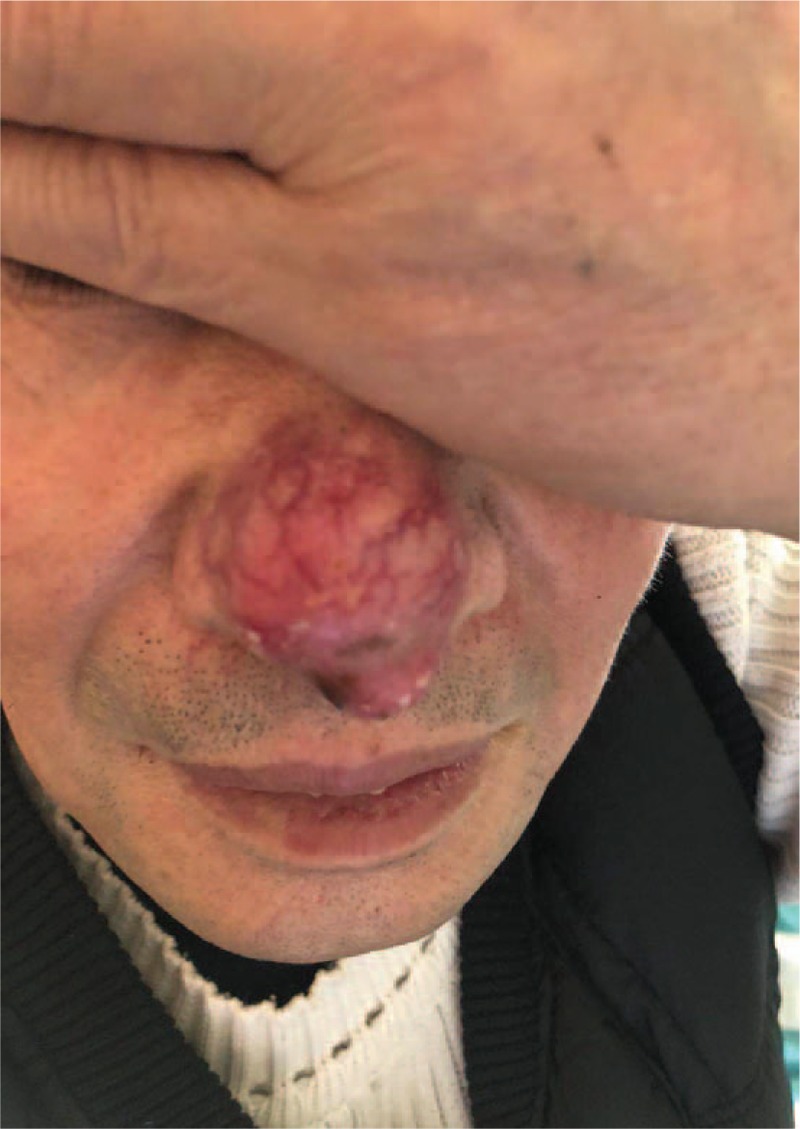
The metastatic lesion on the patient's nasal tip. A round, painless erythematous nodule measuring 3.0 × 3.0 cm with a basal bulge could be observed on the nasal tip, with cutaneous capillary congestion and black hyperpigmentation.

**Figure 2:**
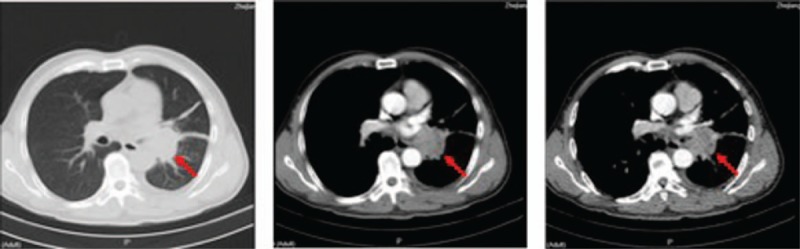
Enhanced CT scan confirmed the existence of neoplasm in the left lower lobe. Red arrows indicate the location of the tumor.

**Figure 3:**
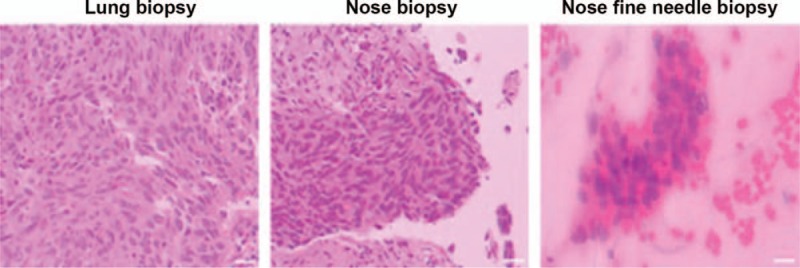
The pathology of lung and nasal tip biopsy, both of which were confirmatory for low-grade differentiated squamous cell carcinoma. Scale bar: 50 μm.

**Figure 4:**
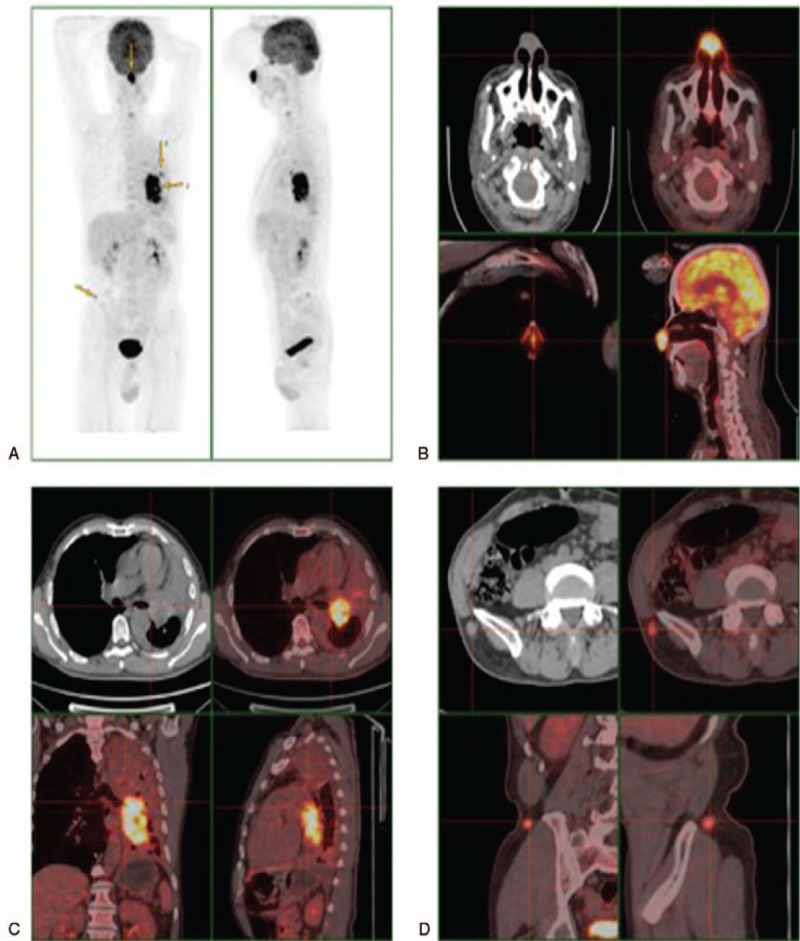
PET-CT scan of the patient, which revealed the nasal tip and suspected buttock metastasis of primary lung cancer. A, whole body scan; B–D, skull, lung, and buttocks scan, respectively, hypermetabolic activities were seen in these areas.

A 62-year-old, non-smoking, Chinese male peasant was admitted to our hospital with complaints of repeated cough and white sputum for 6 months. And he also complained of a mass on his nose tip for nearly 4 months, which was increasing progressively during this period but without obvious pain, itching or other symptoms. On physical examination, a round, painless erythematous nodule measuring 3.0 × 3.0 cm with a basal bulge was observed on his nasal tip, with no signs of ulcer, rather only cutaneous capillary congestion and black hyperpigmentation (Fig. 1). No other skin or limb nodules were seen; further, neither the submandibular nor neck areas showed palpable lymph nodes. Rhonchi and moist rales could be heard in the left lower lung lobe. Laboratory examinations revealed elevated carbohydrate antigen 125 (CA-125; 228.6 U/ml, normal range: 0–35 U/ml) and C-reactive protein (CRP; 67.7 mg/L, normal range: 0–4 mg/L); while tests for blood counts and liver, kidney, and coagulation profiles were normal. After admission, an enhanced chest computed tomography (CT) was performed, which revealed occlusion of the left main bronchus due to a left hilar pulmonary mass (Fig. 2). A followed bronchoscopy and biopsy were carried out 4 days after the CT scan, which revealed that the left main bronchial lumen was severely stenotic owing to the neoplasm. Simultaneously, a biopsy of the nasal tip neoplasm was obtained. Poorly differentiated squamous cell carcinoma was confirmed by pathological examination in both specimens (Fig. 3). However, no positive genetic mutation was found in the sequencing result of the lung cancer specimen (Table 1), which implied that commercially available targeted drug delivery could not be used for treatment. Furthermore, immunohistochemistry (IHC) showed that the nasal tip specimen was highly consistent with the IHC result of the lung specimen (Table 2); this implied that the nasal neoplasm was likely originated from the primary lung cancer. Additionally, ^18^F-fluorodeoxyglucose positron emission tomography (PET)-CT imaging showed hypermetabolic activities in the lower lobe of the left lung, nose, and buttocks; however, no sign of hypermetabolic activities was observed in the brain or bones, which implied that no metastasis existed in these areas (Fig. 4). Finally, this patient was considered as lung squamous carcinoma, T2NxM1b, staging IVa. Given the metastases due to lung cancer, this patient was unsuited for surgery, he also refused the systemic chemotherapy, instead he chose to be discharged soon after PET-CT scan, and to receive conservative and symptomatic treatment with traditional Chinese medicine at home. Unfortunately, the patient died 6 months after the diagnosis.

**Table 1:**
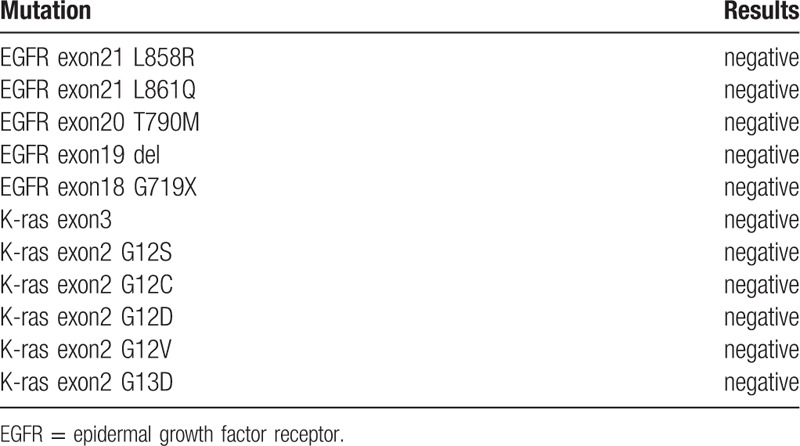
Sequencing results of the patient's lung cancer specimen.

**Table 2:**
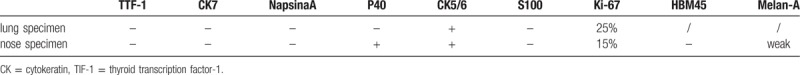
Results of immunohistochemistry staining of the lung and nasal specimen.

## 3 Discussion and conclusion

The skeletal and central nervous systems are the most common distal metastatic sites in patients with lung cancer. Cutaneous metastasis is typically rare and might appear as lesions anywhere on the cutaneous surface.^[9]^ Cases of buttock metastasis are still rarer,^[10–14]^ and in these cases, solitary or multiple painless nodules is the most common manifestation, with no other typical symptoms at presentation.^[15]^ Multiple lesions are usually grouped and show rapid growth, with occasional necrosis or ulceration.^[9]^ In our patient, there was no pain or ulceration accompanied with the nasal neoplasm, and hence, differential diagnosis was needed to rule out other conditions such as rhinophyma, rosacea, furuncle, and hemangioma. Additionally, a biopsy of the suspicious skin lesion was helpful in identifying the histological type. For the buttock lesion, no positive finding on physical examination implied the existence of metastasis, which highlights the critical value of PET-CT scan in cancer patients’ diagnosis and evaluation.

Lung cancer metastasis may occur via 4 routes: hematogenous, lymphatic, endobronchial, and direct spread. The mechanism of metastasis in distal soft tissues such as skin and skeletal muscle remains unclear; hematogenous spread is still considered the primary pathway for distal dissemination.^[16]^ Furthermore, there was no significant difference in the incidence of skin metastasis among all histologic types of lung cancer.^[17]^ However, there is a controversy that adenocarcinoma is the most common type of metastasis from lung cancer, followed by squamous cell carcinoma.^[18]^ Direct invasion might occur in cases of spinal epidural metastasis,^[19]^ and occasionally, after bronchoscopic intervention.^[20]^ In our patient, no palpable lymph nodes could be found in the submandibular and neck areas, which might imply that the nasal metastasis was formed through a hematogenous route.

The presence of soft tissue metastasis in lung cancer may affect staging and prognosis, usually it was regarded as a sign of grave prognostic indicator, with a poorly differentiated and advanced primary cancer.^[21,22]^ Patients presenting with soft tissue metastasis secondary to lung cancer have a maximum median survival of 4 months.^[16]^ The choice of treatment for patients with metastatic NSCLC depends on many factors including comorbidity, histology, and molecular genetic features of the cancer; standard treatment options for metastatic NSCLC may include palliative external radiation therapy, combination chemotherapy, and combination chemotherapy and targeted therapy.^[23]^ In our case, this patient had multiple soft tissues metastasis; in other words, at the time of diagnosis, he was unsuited for surgical resection of solitary metastases and primary tumors. Sequencing results showed no positive mutation in his specimen, which implied that no targeted therapy could be employed. Although he refused the systemic chemotherapy and died 6 months after the diagnosis, his survival period was somewhat longer than the maximum median survival of 4 months as mentioned above,^[16]^ and there was the likelihood of extended survival if systemic treatments were administered. This case report highlights that for multiple skin nodules or rapidly growing nodules, the possibility of skin metastasis of the primary cancers should be taken into account. Early diagnosis and proper treatment strategy may prolong patient survival.

In conclusion, when a patient presents with a single tumor or multiple tumors in the soft tissues, with or without known primary cancer, clinicians should be alert to the possibility primary cancer metastases, and a biopsy of the suspicious skin lesion should be obtained to aid the histological identification of primary cancer. PET-CT scan could be used as an effective tool for diagnosis and evaluation.

## Acknowledgments

The authors would like to thank the patient and family for their contribution to this study.

## Author contributions

TCS and ZXX designed the study and wrote the manuscript. CJL and YFF participated the study and collected the data. All authors read and approved the manuscript.
